# Supplementary material for: Test-retest reliability and validity of the Importance of Olfaction Questionnaire in Denmark
Source: PLoS One. 2024 Jan 19;19(1):e0269211. doi: 10.1371/journal.pone.0269211 (PMC10798468; doi:10.1371/journal.pone.0269211)
Supplement: S1 Table — The IO-Q has 20-items and reports are made on a 4-point Likert scale that is scored 0 for complete disagreement and 3 for complete agreement. The items are separable into four categories (association (Ass), consequence (Con), application (App), and aggravation (Agg)). Each of the subscales are made of 6 items except Agg with only 2. The Scale column is not printed when the questionnaire is presented to participants. (DOCX) [file pone.0269211.s001.docx]

**Individuel vigtighed af lugtesansen**

Dette spørgeskema omhandler hvilken rolle din lugtesans har i din dagligdag. Besvar venligst alle spørgsmålene spontant, der er ikke nogen rigtige eller forkerte svar.

|  | Scale | Helt  enig | Overvejende enig | Overvejende uenig | Helt uenig |
| --- | --- | --- | --- | --- | --- |
| En persons lugt spiller en rolle i min beslutning om jeg kan lide ham/hende. | Ass |  |  |  |  |
| Jeg lugter til mad for at finde ud af om det er fordærvet eller ej. | App |  |  |  |  |
| Jeg lugter til maden før jeg spiser det. | App |  |  |  |  |
| Forestil dig at du er på et museum. Der er et tilbud om at få yderligere lugteindtryk til udstillingen med henblik på at fremhæve det overordnede indtryk for 15 kr.  Ville du tage imod dette tilbud? | Con |  |  |  |  |
| Når jeg ikke kan lide duften af en shampoo, køber jeg den ikke. | Con |  |  |  |  |
| Når jeg lugter til lækker mad bliver jeg sulten. | Ass |  |  |  |  |
| Uden min lugtesans ville livet være meningsløst. | Agg |  |  |  |  |
| Jeg forsøger at finde ud af hvor en lugt kommer fra når jeg lugter noget. | Con |  |  |  |  |
| Jeg bliver hurtigt generet af lugte i mine omgivelser. | Ass |  |  |  |  |
| Bestemte lugte kan øjeblikkeligt udløse mange minder. | Ass |  |  |  |  |
| Før jeg drikker the/kaffe, lugter jeg bevidst til det. | App |  |  |  |  |
| Når jeg køber tomater er jeg opmærksom på deres lugt. | App |  |  |  |  |
| Hvis min partner har en væmmelig lugt, undgår jeg at kysse ham/hende. | Con |  |  |  |  |
| Bestemte lugte kan øjeblikkeligt udløse stærke følelser. | Ass |  |  |  |  |
| Jeg lugter til mit tøj for at vurdere om det trænger til at blive vasket. | App |  |  |  |  |
| Når der er en væmmelig lugt på kontoret eller i en kollegas hjem, forlader jeg rummet så hurtigt som muligt. | Con |  |  |  |  |
| Bestemte lugte kan stimulere min fantasi. | Ass |  |  |  |  |
| For mig er det mere vigtigt at kunne lugte end at kunne se eller høre. | Agg |  |  |  |  |
| Sommetider lugter jeg til en person (f.eks. min partner eller mit barn) for at vurdere om han/hun har drukket eller røget. | App |  |  |  |  |
| Jeg kan ikke gå forbi velduftende stearinlys i en butik uden at købe et. | Con |  |  |  |  |
