# Supplementary material for: Test-retest reliability and validity of the Importance of Olfaction Questionnaire in Denmark
Source: PLoS One. 2024 Jan 19;19(1):e0269211. doi: 10.1371/journal.pone.0269211 (PMC10798468; doi:10.1371/journal.pone.0269211)
Supplement: S2 Table — List of results in other studies [9, 20, 21]. Note that these studies differ either in how the IOQ was scored (IOQ18 using only the total App, Ass and Con while leaving out Agg) or in terms of the culture of the studies population (Asian or American rather than European). (DOCX) [file pone.0269211.s002.docx]

| **Study and measure** | **N** | **Mean** | **SD** |
| --- | --- | --- | --- |
| Present study (Danish): IOQ18_1 | 179 | 31.39 | 6.29 |
| Present study (Danish): IOQ18_2 | 179 | 33.37 | 5.70 |
| Murr et al. (2018) (German females aged below 25): IOQ18 | 73 | 29.52 | 9.36 |
| Murr et al. (2018) (German males aged below 25): IOQ18 | 62 | 25.56 | 9.54 |
| Murr et al. (2018) (German females aged 25-50): IOQ18 | 66 | 23.04 | 10.44 |
| Murr et al. (2018) (German males aged 25-50): IOQ18 | 73 | 24.12 | 9.72 |
| Liu et al. (2020) (Austrian): IOQ18 | 50 | 35 | 6.1 |
|  |  |  |  |
| Present study (Danish) IOQ20_1: IOQ20 | 179 | 32.93 | 6.78 |
| Present study (Danish) IOQ20_2: IOQ20 | 179 | 34.71 | 6.25 |
| Li et al. (2022) (Chinese females aged 18-29): IOQ20 | 1204 | 38.12 | 7.5 |
| Li et al. (2022) (Chinese males aged 18-29): IOQ20 | 699 | 37.1 | 8 |
| Li et al. (2022) (Indian): IOQ20 | 3172 | 39.95 | 10.98 |
| Li et al. (2022) (American):IOQ20 | 4026 | 36.59 | 9.73 |
