# Supplementary figures and images for: Test-retest reliability and validity of the Importance of Olfaction Questionnaire in Denmark
Source: PLoS One. 2024 Jan 19;19(1):e0269211. doi: 10.1371/journal.pone.0269211 (PMC10798468; doi:10.1371/journal.pone.0269211)

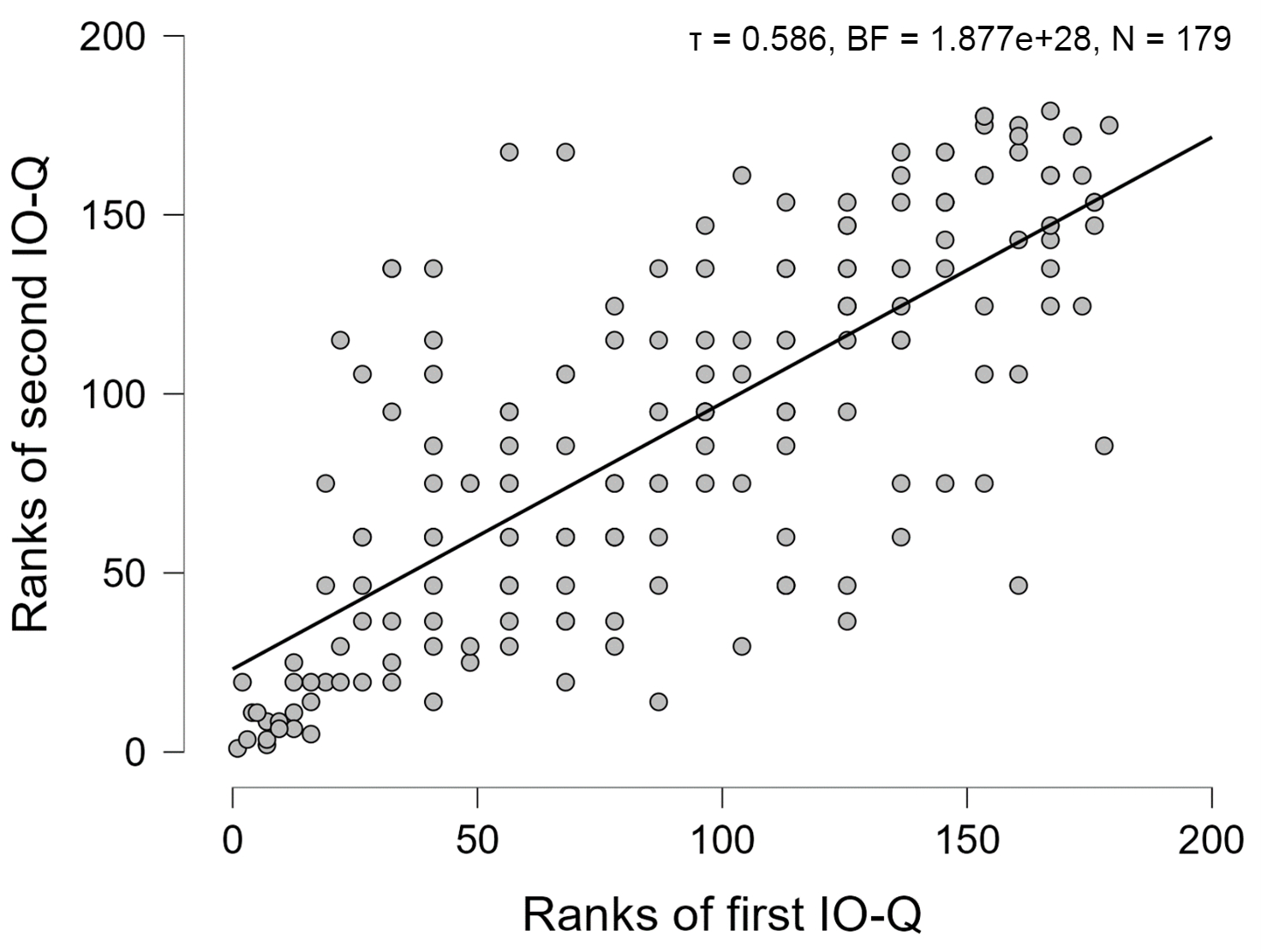

Supplement: S1 Fig — Non-parametric correlational scatterplot between the total score of the first test (Ranks of first IO-Q) and the second test (Ranks of second IO-Q). Notice the extreme evidence for positive correlation. Note that data points may overlap. (TIFF) [file pone.0269211.s003.tiff]
